# Recurrent Unilateral Vulvar Syringoma Mimicking Treatment-Resistant Condyloma Acuminata: A Case Report and Review of the Literature

**DOI:** 10.3390/healthcare14172807

**Published:** 2026-09-01

**Authors:** Saeed Baradwan, Mohammad Alyafi, Haneen Al-Maghrabi, Sumaih Shinawi

**Affiliations:** 1 Department of Obstetrics and Gynecology, King Faisal Specialist Hospital and Research Center, Jeddah 23433, Saudi Arabia; 2 Department of Pathology and Laboratory Medicine, King Faisal Specialist Hospital and Research Center, Jeddah 23433, Saudi Arabia

**Keywords:** vulvar syringoma, condyloma acuminata, vulvar lesion, eccrine neoplasm, vulvar pruritus

## Abstract

**Background:** Vulvar syringoma is a rare benign adnexal neoplasm originating from eccrine sweat ducts. Although syringomas most commonly occur in the periorbital region, vulvar involvement is uncommon and frequently underrecognized. Due to its nonspecific clinical appearance, vulvar syringoma may mimic more common vulvar conditions, resulting in delayed diagnosis and repeated ineffective treatments. **Case Presentation:** A 41-year-old multiparous woman presented with a several-year history of recurrent pruritic papular lesions involving the right vulva. The lesions were repeatedly diagnosed as condyloma acuminata and treated with topical imiquimod, cryotherapy, carbon dioxide laser ablation, and local excision, without sustained improvement. Persistent symptoms and recurrence prompted repeat surgical excision. Histopathological examination revealed a well-circumscribed dermal lesion composed of multiple small eccrine ducts, cysts, and epithelial cords embedded within a dense sclerotic fibrotic stroma. The ducts were lined by two layers of bland cuboidal epithelial cells and demonstrated characteristic comma-shaped and tadpole-like configurations, confirming the diagnosis of vulvar syringoma. No cytologic atypia, increased mitotic activity, dysplasia, or malignancy was identified. The postoperative course was uncomplicated, with satisfactory wound healing and symptomatic improvement during follow-up. **Conclusions:** Vulvar syringoma should be considered in the differential diagnosis of persistent or treatment-resistant vulvar papules, particularly when lesions recur despite conventional therapy for presumed condyloma acuminata or other benign vulvar disorders. Early biopsy and histopathological evaluation remain essential for establishing an accurate diagnosis, preventing unnecessary interventions, and guiding appropriate management.

## 1. Introduction

Syringoma is a benign adnexal neoplasm of eccrine sweat duct origin that most commonly occurs in the periorbital region. Genital involvement is uncommon, and vulvar localization represents a particularly rare clinical presentation [1,2]. Vulvar syringomas typically present as multiple small, skin-colored to yellow-brown papules and may be associated with chronic vulvar pruritus and cosmetic concerns [2,3].

Because of their rarity and nonspecific clinical appearance, vulvar syringomas may be mistaken for more common vulvar disorders, particularly condyloma acuminata and other benign vulvar lesions [3,4]. Consequently, affected patients may undergo repeated empirical treatments before histopathological examination establishes the definitive diagnosis. We report a case of recurrent unilateral vulvar syringoma that was repeatedly treated as treatment-resistant condyloma acuminata over an 8-year period before the definitive diagnosis was established through wide local excision and histopathological evaluation.

## 2. Case Presentation

A 41-year-old multiparous woman was referred to the gynecology clinic with an approximately 8-year history of recurrent papular lesions involving the right vulva. The lesions were associated with intermittent vulvar pruritus and increasing cosmetic concern. During the first 4 years of her illness, the lesions were clinically presumed to represent condyloma acuminata and were treated sequentially with topical imiquimod, repeated cryotherapy sessions, carbon dioxide (CO_2_) laser ablation, and local surgical excision. Despite these interventions, the lesions persisted and subsequently recurred. A previous histopathological examination from an earlier excision was reportedly interpreted as benign neural proliferation and did not establish a definitive diagnosis. The original histopathological specimen was not available for re-evaluation, and this information was based solely on the previous pathology report. Following recurrence after the previous treatments, the patient was recently referred for further gynecological evaluation and definitive management.

Physical examination revealed multiple clustered, flesh-colored papules predominantly involving the right labia majora, measuring approximately 5 × 4 cm in total area. No suspicious features such as pigmentation, ulceration, bleeding, or inguinal lymphadenopathy were identified. The remainder of the gynecological examination was unremarkable.

Given the persistent recurrence and resistance to conventional treatments, wide local excision of the affected vulvar tissue was performed for diagnostic confirmation and therapeutic management. The procedure was completed without intraoperative complications.

**Figure 1:**
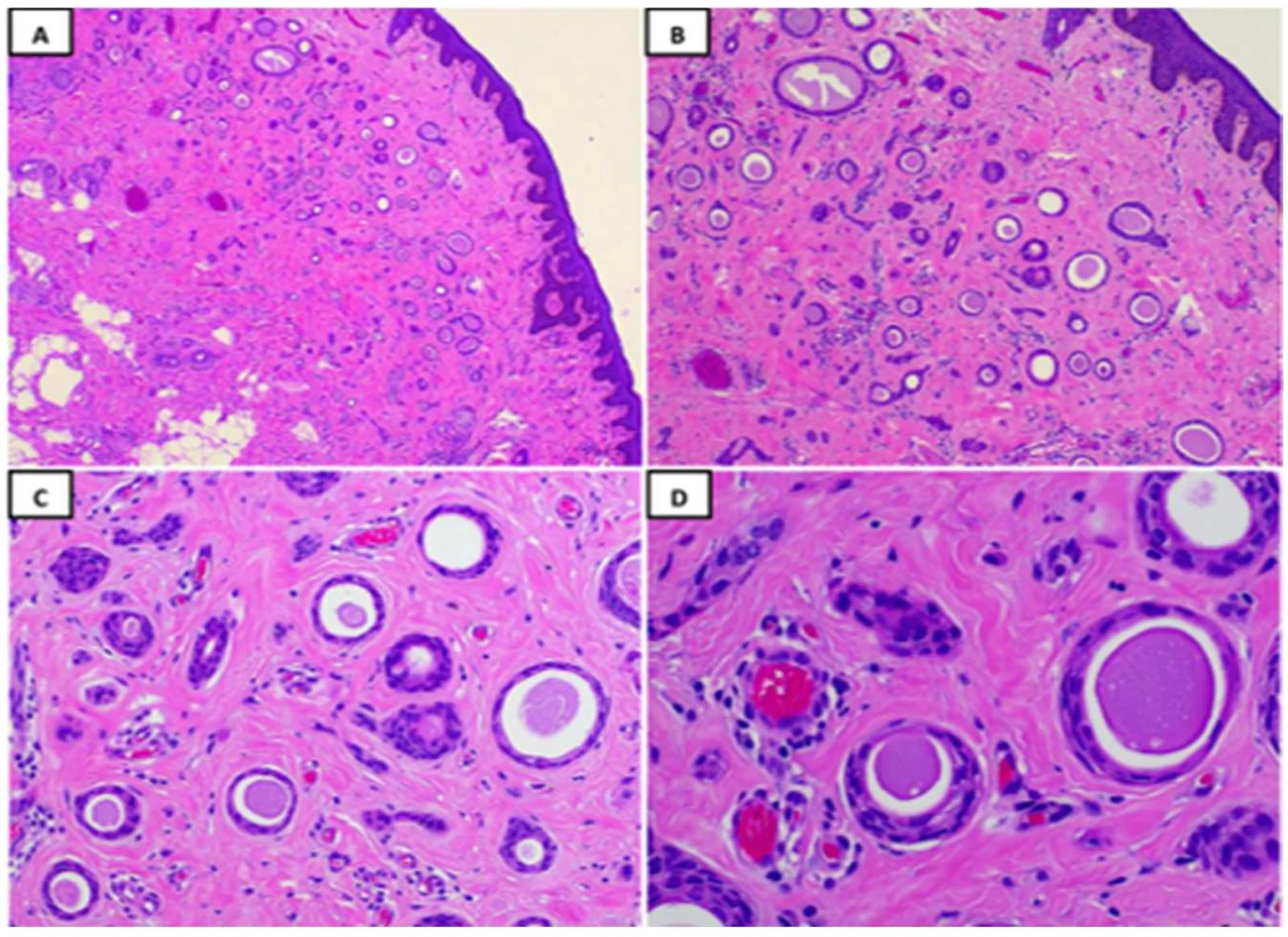
Histopathological examination of vulvar syringoma using hematoxylin and eosin (H&E) staining. (**A**) Circumscribed proliferation of bilayered ductular structures involving superficial dermis (H&E, ×2). (**B**) Ductular structures lined by two layers of bland epithelial cells embedded within densely fibrotic stroma (H&E, ×4). (**C**) Benign proliferation of round-to-comma-shaped ductules (H&E, ×10). (**D**) No significant cytologic atypia or mitotic figures are identified (H&E, ×20).

Histopathological examination revealed a dermal, well-circumscribed lesion composed of cysts, ducts, and cords lined by bland cuboidal epithelial cells. Some ducts contained granular basophilic material, with characteristic small eccrine ductal structures embedded within a sclerotic fibrotic stroma (Figure 1). No significant cytologic atypia, increased mitotic activity, necrosis, or invasive growth pattern was observed. The histopathological findings confirmed the diagnosis of vulvar syringoma. Surgical margins were free of syringoma, and no dysplastic changes were identified.

The postoperative course was uneventful. The patient remained clinically stable with satisfactory pain control using standard postoperative analgesia. No wound infection, hematoma formation, wound dehiscence, urinary dysfunction, or postoperative bleeding was observed. At the 6-month follow-up, the patient reported improvement in vulvar pruritus, and no clinical evidence of recurrence was identified.

### Literature Search

A focused literature search was conducted to identify previously published cases and case series of vulvar syringoma and to provide a comprehensive comparison of the clinical presentation, histopathological findings, differential diagnosis, treatment, and outcomes with the present case. The search was performed in PubMed/MEDLINE, Scopus, Web of Science, and Google Scholar from database inception to 30 June 2026. The search strategy used combinations of the following keywords and Medical Subject Headings (MeSH) terms: “vulvar syringoma,” “vulval syringoma,” “syringoma of the vulva,” “genital syringoma,” “syringoma” AND “vulva,” and “syringoma” AND “pruritus vulvae.” The reference lists of relevant articles were also manually screened to identify additional eligible publications. Articles were considered eligible if they reported histopathologically confirmed vulvar syringoma and provided relevant clinical, pathological, therapeutic, or follow-up information. Case reports, case series, and clinicopathological studies were included. Articles describing syringoma at non-vulvar sites without relevant vulvar findings, duplicate publications, and reports without sufficient information to confirm vulvar involvement were excluded. No restrictions were applied based on patient age or publication year. Data extracted from eligible reports included patient demographics, clinical presentation, lesion distribution, symptoms, initial diagnosis, histopathological findings, treatment modality, follow-up duration, and reported outcome.

## 3. Discussion

Vulvar syringoma is a rare benign adnexal neoplasm of eccrine sweat duct origin involving the vulva, an uncommon site for syringoma. Although syringomas most frequently occur in the periorbital region, vulvar localization is distinctly uncommon and may therefore remain underrecognized [1,2,3]. Clinically, vulvar syringomas usually present as multiple small, flesh-colored to yellow-brown papules, typically involving the labia majora, and are frequently associated with chronic vulvar pruritus, although asymptomatic lesions have also been described [2,3,4]. Because of their rarity and nonspecific clinical appearance, vulvar syringomas may be mistaken for more common vulvar disorders, including condyloma acuminata, Fox–Fordyce disease, epidermal inclusion cysts, angiokeratomas, steatocystoma multiplex, and other benign vulvar lesions [1,3,4].

The present case illustrates the diagnostic challenge posed by vulvar syringoma. Our patient experienced a prolonged clinical course during which the lesions were repeatedly diagnosed as condyloma acuminata and treated with topical imiquimod, cryotherapy, carbon dioxide laser ablation, and repeated local excision without sustained clinical improvement. Similar diagnostic delays have been described in previous reports and may reflect the close clinical resemblance of vulvar syringoma to viral genital warts and other benign vulvar conditions [1,3,4]. Persistent or recurrent vulvar papules that fail to respond to conventional therapy should therefore prompt the reconsideration of the diagnosis and early biopsy for definitive histopathological evaluation [1,3,4].

The broad clinical differential diagnosis of vulvar syringoma may contribute substantially to diagnostic delay because its appearance may overlap with several benign vulvar disorders and, less commonly, malignant adnexal neoplasms. Principal differential diagnoses include condyloma acuminata, Fox–Fordyce disease, epidermal inclusion cysts, steatocystoma multiplex, angiokeratomas, lymphangioma circumscriptum, and other papular vulvar disorders [1,3,4]. Microcystic adnexal carcinoma is an important histopathological differential diagnosis because of its potential similarity to syringoma, particularly in lesions with deeper extension [5]. Histopathological examination distinguishes syringoma by demonstrating numerous small eccrine ducts lined by two layers of bland cuboidal epithelial cells within a dense fibrotic or sclerotic stroma, with characteristic comma-shaped or tadpole-like epithelial extensions [4,5]. The key clinical and histopathological features distinguishing these entities are summarized in Table 1.

The clinicopathological characteristics, management, and outcomes of previously reported cases are summarized in Table 2 [1,2,3,4,5,6,7,8,9,10,11,12]. Comparison with the present case highlights the uncommon unilateral distribution of vulvar syringoma, the potential for initial clinical misdiagnosis as condyloma acuminata or other benign vulvar disorders, and the importance of histopathological examination in establishing the definitive diagnosis [1,3,4]. Most reported patients were young or middle-aged women and presented with vulvar papules, frequently accompanied by pruritus [1,2,3,4,6,9,11,12]. The clinical appearance of vulvar syringoma may overlap with several common vulvar disorders, which can contribute to diagnostic uncertainty and delay [1,3,4]. Similarly to some previously reported cases, our patient underwent several unsuccessful treatment modalities before the correct diagnosis was established through histopathological examination. These observations underscore that persistent or recurrent vulvar papules that do not respond to conventional therapy should prompt the reconsideration of the diagnosis and timely biopsy to avoid unnecessary interventions and further diagnostic delay [1,3,4].

Histopathological examination remains essential for the definitive diagnosis of vulvar syringoma. Classic microscopic findings include numerous small ducts, tubules, and epithelial cords embedded within a dense fibrotic or sclerotic stroma. The ducts are characteristically lined by two layers of bland cuboidal epithelial cells and may demonstrate comma-shaped or tadpole-like epithelial extensions [4,5,9]. Cytologic atypia, increased mitotic activity, necrosis, and destructive deep infiltration are generally absent, helping to distinguish syringoma from malignant adnexal neoplasms, including microcystic adnexal carcinoma [4,5]. Immunohistochemical studies may provide additional information in diagnostically challenging cases; however, estrogen and progesterone receptor expression has not been consistently demonstrated in vulvar syringoma. In the largest clinicopathological series, estrogen and progesterone receptors were negative in all 15 tumors evaluated [4].

The pathogenesis of vulvar syringoma remains incompletely understood. Hormonal influences have been proposed because the exacerbation of vulvar pruritus or lesion activity has been reported during menstruation and pregnancy [2,6]. However, immunohistochemical studies have not consistently demonstrated estrogen or progesterone receptor expression, and the precise role of hormonal factors in the development or progression of vulvar syringoma remains uncertain [4,6]. Therefore, hormonal influence should currently be regarded as a possible modifying factor rather than an established etiological mechanism.

Dermoscopy has recently been described as a useful non-invasive adjunct in the assessment of syringoma. Reported dermoscopic findings include whitish or yellowish homogeneous structures, white dots, and, in the vulvar variant, glittering yellow-whitish round structures with dotted or short linear vessels [13]. Although these findings may increase clinical suspicion, they are not pathognomonic and may overlap with other benign papular lesions. Consequently, histopathological examination remains necessary for definitive diagnosis, particularly in persistent or treatment-resistant vulvar lesions.

The management of vulvar syringoma remains challenging because no single treatment has demonstrated consistently superior long-term efficacy. Treatment is generally considered for symptomatic lesions or significant cosmetic concerns. Reported therapeutic options include surgical excision, carbon dioxide laser vaporization, electrosurgery, cryotherapy, dermabrasion, topical atropine, and topical retinoids, although the available evidence is largely derived from case reports and small case series [4,8,10,12]. Carbon dioxide laser treatment has demonstrated favorable symptomatic outcomes in patients with severe pruritus, while topical adapalene has also been reported to improve symptoms in a patient with long-standing vulvar syringoma [4,8,12]. Recurrence may occur following treatment, particularly after superficial or incomplete destruction, because syringomas may extend into the deeper dermis [5]. Accordingly, treatment should be individualized according to lesion extent, symptoms, cosmetic concerns, and the risk of recurrence.

An additional noteworthy feature of the present case is the unilateral distribution of the lesions. Vulvar syringomas are often described as multiple and may be bilateral, whereas unilateral involvement is less commonly reported [1,2,3,4]. This unusual distribution, together with the prolonged clinical course and repeated recurrence following treatment, contributed to the initial clinical impression of treatment-resistant condyloma acuminata. Furthermore, our patient had undergone a previous biopsy that reportedly demonstrated benign neural proliferation without establishing a definitive diagnosis. This history further underscores the importance of adequate tissue sampling and careful histopathological evaluation in recurrent or diagnostically challenging vulvar lesions.

As with all single case reports, these findings may not be generalizable to the broader population. An additional limitation is that preoperative clinical photographs of the vulvar lesions were not obtained because the patient had undergone multiple previous treatments before referral to our institution. Consequently, only postoperative histopathological images were available to illustrate the diagnosis. Nevertheless, the detailed clinical history, histopathological findings, and available follow-up provide sufficient evidence to support the diagnosis and the educational value of this case. This case demonstrates that vulvar syringoma may closely mimic condyloma acuminata and other common vulvar disorders. Increased awareness among gynecologists, dermatologists, and pathologists may facilitate earlier diagnosis and reduce unnecessary or ineffective treatments. Persistent or recurrent vulvar papules that fail to respond to conventional therapy should prompt timely histopathological evaluation to establish the correct diagnosis and guide appropriate management.

## 4. Conclusions

Vulvar syringoma is a rare benign eccrine adnexal neoplasm that should be considered in the differential diagnosis of persistent or recurrent vulvar papules, particularly when lesions are unresponsive to conventional treatment for condyloma acuminata. Histopathological examination remains essential for establishing the diagnosis and preventing unnecessary or repeated therapeutic interventions. The increased awareness of this uncommon entity among clinicians may facilitate earlier diagnosis, appropriate management, and improved patient outcomes.

## Figures and Tables

**Table 1:** Differential diagnosis of vulvar syringoma and distinguishing clinical and histopathological features.

Condition	Typical Clinical Features	Histopathological Features	Distinguishing Features from Vulvar Syringoma
**Vulvar syringoma**	Multiple small, flesh-colored to yellowish papules; often pruritic; usually bilateral but may be unilateral; slow-growing	Numerous small eccrine ducts lined by two layers of cuboidal epithelial cells within a dense sclerotic stroma; characteristic comma-shaped (“tadpole”) ducts	Presence of characteristic eccrine ductal structures within a fibrotic stroma confirms the diagnosis.
**Condyloma acuminata**	Multiple exophytic or verrucous papules caused by HPV; may be asymptomatic or pruritic	Papillomatosis, acanthosis, hyperkeratosis, and koilocytosis	Lacks eccrine ductal differentiation and comma-shaped ducts.
**Epidermal inclusion cyst**	Solitary, slow-growing, subcutaneous nodule; may become inflamed	Cyst lined by stratified squamous epithelium filled with lamellated keratin	True cystic lesion without eccrine duct proliferation.
**Fox–Fordyce disease**	Intensely pruritic follicular papules involving apocrine-rich areas; commonly affects young women	Follicular hyperkeratosis with dilated apocrine ducts and perifollicular inflammation	Involves apocrine glands rather than eccrine ducts; inflammatory changes are prominent.
**Lichen simplex chronicus**	Thickened, hyperpigmented plaques associated with chronic scratching and severe pruritus	Hyperkeratosis, acanthosis, and chronic inflammatory infiltrate	Reactive epidermal changes without adnexal duct proliferation.
**Vestibular papillomatosis**	Symmetric, soft, pink papillae on the vestibule; normal anatomical variant	Normal squamous epithelium with fibrovascular cores	Benign anatomical variant lacking ductal or adnexal proliferation.
**Steatocystoma multiplex**	Multiple dermal cystic nodules, often involving the trunk, axillae, or genital region	Sebaceous duct cysts lined by stratified squamous epithelium with sebaceous glands in the cyst wall	Sebaceous differentiation rather than eccrine ductal proliferation.
**Microcystic adnexal carcinoma**	Rare, indurated, locally infiltrative lesion; slow-growing	Infiltrative cords and ducts extending into deep dermis with perineural invasion	Cytologic blandness may overlap, but infiltrative growth and perineural invasion distinguish malignancy from benign syringoma.

**Table 2:** Clinical characteristics, symptoms, location, initial clinical diagnosis, treatment, and outcomes of selected reported cases of vulvar syringoma.

Author (Year)	Age (Years)	Presentation	Symptoms	Location	Initial Clinical Diagnosis	Treatment	Outcome
Young et al. (1980) [1]	Not reported	Multiple vulvar papules	Pruritus	Vulva	Differential diagnosis included benign vulvar disorders	Histopathological examination	Diagnosis confirmed; follow-up not reported
Gerdsen et al. (2002) [2]	27	Multiple firm, skin-colored papules, up to 5 mm	Periodic vulvar pruritus, aggravated during menstruation	Labia majora extending to both groins	Condyloma acuminata and other benign lesions considered	Histopathological confirmation; laser treatment discussed	Diagnosis confirmed; treatment benefit limited; follow-up not reported
Miranda et al. (2002) [3]	29	Small condylomatous-type vulvar lesion	Vulvar itching and burning	Vulva	Condyloma acuminata	Biopsy	Syringoma confirmed histologically; follow-up not reported
Huang et al. (2003) [4]	21–60	Multiple flesh-colored or brownish papules	Pruritus in 13/18 patients	Vulva, commonly bilateral	Variable clinical diagnoses	Various treatments; CO_2_ laser vaporization in symptomatic patients	Lesions resolved and pruritus subsided in treated patients
Turan et al. (1996) [6]	Not reported	Vulvar syringoma with pregnancy-associated exacerbation	Pruritus worsened during pregnancy	Vulva	Not reported	Not clearly reported	Symptoms exacerbated during pregnancy; long-term follow-up not reported
Dereli et al. (2007) [7]	23	Multiple syringomatous papules	Not reported	Vulva	Not reported	Not reported	Follow-up not reported
Ercan et al. (2012) [8]	35	Multiple small vulvar papules present for approximately 3 years	Intermittent pruritus	Vulva	Not reported	Laser vaporization	No recurrence at 1-month follow-up; patient reported satisfaction
Nibhoria et al. (2014) [9]	46	Multiple vulvar papules	Itching and burning for 2 years	Vulva/labia majora	Not reported	Punch biopsy	Histopathological diagnosis confirmed; follow-up not reported
Shalabi et al. (2022) [10]	Not reported	Vulvar syringomatous papules	Vulvar discomfort, burning, and/or pruritus	Vulva	Not reported	Conservative treatment; procedural treatment for refractory disease	Clinical improvement reported; duration of follow-up not reported
George et al. (2022) [11]	45	Vulvar papules associated with facial syringomas	Severe vulvar pruritus	Vulva	Not reported	Medical/conservative management	Diagnosis confirmed clinically and histopathologically; follow-up not reported
Uesugi-Uchida et al. (2022) [12]	Not reported	Long-standing vulvar syringoma with facial involvement	Severe, persistent vulvar pruritus	Vulva and face	Not reported	Topical adapalene	Marked improvement in vulvar pruritus
Regmi et al. (2023) [5]	Not reported	Solitary vulvar syringoma with deep extension	Pain	Vulva	Microcystic adnexal carcinoma considered	Surgical excision	Histopathology confirmed syringoma; follow-up not reported
Present case (2026)	41	Multiple clustered flesh-colored papules, approximately 5 × 4 cm, recurrent over 8 years	Intermittent pruritus and cosmetic concern	Right labia majora	Treatment-resistant condyloma acuminata	Imiquimod, repeated cryotherapy, CO_2_ laser ablation, previous local excision, followed by definitive wide local excision	Histopathologically confirmed vulvar syringoma; at 6-month follow-up, symptomatic improvement and no early recurrence during the available follow-up period

## Data Availability

The original contributions presented in this study are included in the article. Further inquiries can be directed to the corresponding author.
